# Is tea consumption associated with reduction of risk of rheumatoid arthritis? A Swedish case-control study

**DOI:** 10.1186/s13075-021-02583-y

**Published:** 2021-08-07

**Authors:** Helga Westerlind, Ida Palmqvist, Saedis Saevarsdottir, Lars Alfredsson, Lars Klareskog, Daniela Di Giuseppe

**Affiliations:** 1 Clinical Epidemiology Division, Department of Medicine, Solna, Karolinska Institutet, Stockholm, Sweden; 2 Rheumatology Division, Department of Medicine (Solna), Karolinska Institutet, 171 76 Stockholm, Sweden; 3 Faculty of Medicine, School of Health Sciences, University of Iceland, Reykjavik, Iceland; 4 Institute of Environmental Medicine, Karolinska Institutet, Stockholm, Sweden

**Keywords:** Rheumatoid arthritis, Risk, Tea, Diet, ACPA, Smoking

## Abstract

**Background:**

Tea is a popular beverage around the world and has properties that can affect the immune system. The association between tea consumption and the risk of rheumatoid arthritis (RA), a chronic autoimmune disease primarily affecting the joints, is not well studied and results are conflicting.

**Methods:**

We collected data on tea consumption for 2237 incident RA cases diagnosed 2005–2018 and 4661 controls matched on age, sex, and residential area. Tea consumption was classified into no (0 cups/day), irregular (< 1 cup/day), regular (1–2 cups/day), and high (≥ 2 cups/day) consumption, and irregular consumption was used as the reference category. Missing data on tea consumption was classified as no consumers, and sensitivity analyses were performed to test this assumption. Odds ratios (ORs) and 95% confidence intervals (CIs) were calculated using conditional logistic regression, adjusting for smoking, coffee, alcohol, educational level, and body mass index. We also performed stratified analysis on sex, anti-citrullinated autoantibody (ACPA) status, and smoking habits.

**Results:**

Among the cases, we found 57.3% to be ever consumers of tea with 19.7 having a high tea consumption. Corresponding figures for the controls were 58.4% ever drinkers with 22.1% high tea consumers. High tea consumption had an inverse association to the risk of RA compared to irregular consumption [OR = 0.78 (95% CI 0.66–0.92)], but the association lost statistical significance in the adjusted model [adjusted OR (adjOR) = 0.85 (95% CI 0.71–1.01)]. Among non-tea consumers, a protective effect was also observed compared to irregular consumers [adjOR = 0.82 (95% CI 0.70–0.88)], but this association did not withstand sensitivity analysis, possibly due to bias. In the ACPA-positive group and among current smokers, a protective effect of tea consumption was observed among the high tea consumers [adjOR = 0.76 (95% CI 0.62–0.94) and adjOR = 0.60 (95% CI 0.38–0.95), respectively].

**Conclusions:**

This study suggests a protective effect of high consumption of tea, among smokers and for ACPA-positive RA.

**Trial registration:**

Not applicable

**Supplementary Information:**

The online version contains supplementary material available at 10.1186/s13075-021-02583-y.

## Background

Rheumatoid arthritis (RA) is a chronic inflammatory disease with prevalence varying from 0.1 to 1% across the world [1]. The disease is characterized by swollen synovial joints and causes pain and disability [2]. The heritability has been estimated to be around 40% [3]. The etiology of RA is still not clear, and, among environmental factors, only smoking is a well-established risk factor for seropositive RA.

In recent years, diet has gained attention for its potential immunomodulatory effects. The spotlight has been directed to, among others, alcohol [4], omega-3 polyunsaturated fatty acids [5], and the Mediterranean diet [6, 7]. The Mediterranean diet’s protective effects may be attributable to not only its high degree of flavonoids but also its composition of fatty acids which play a role in the immune-mediated inflammatory responses [8].

After water, tea is the most popular drink in the world, and just like the Mediterranean diet, all kinds of tea are rich in polyphenols, which are antioxidants that may reduce the risk of inflammatory diseases such as RA [9]. However, studies on tea consumption and the risk of developing RA have thus far shown inconclusive results. Results from the Nurse’s Health Study showed no association [10], while in the Iowa Women’s Health Study, tea was inversely associated with RA [11] and in the Women’s Health Initiative Observational Study ever tea consumption was associated with an increased risk of RA [12]. Moreover, a case-control study from the UK conducted in both men and women found no association between tea consumption and polyarthritis [13], while a case-control study from Iran showed an inverse association with green tea [14]. A meta-analysis, based only on three of the cited studies [10, 11, 13], concluded that there was no association between tea consumption and the risk of developing RA [15].

The aim of the present study was to investigate the association between tea consumption and the risk of newly diagnosed RA in a large population-based case-control study of RA, the Epidemiological Investigation of RA (EIRA) study.

## Methods

### Study population

Data from the EIRA study, a population-based case-control study, have been used for this project [16]. The EIRA study includes newly diagnosed RA cases from selected rheumatologic units in central and southern parts of Sweden since 1996. RA cases have been diagnosed by a rheumatologist according to either the American College of Rheumatology (ACR) 1987 criteria or the 2010 ACR/European League Against Rheumatism (EULAR) classification criteria for RA. Each case was randomly matched to two controls selected from the total population register, based on age, sex, and residential area at date of the index patient’s diagnosis. The participation rate was 90% for cases and 69% for controls.

Cases and controls filled in a comprehensive questionnaire on lifestyle factors, educational level, and comorbidities, including a 124-item food frequency questionnaire (FFQ). Since the FFQ was introduced in EIRA in 2005, the current study includes only EIRA participants from October 2005 until May 2018. Participants with incomplete FFQ were excluded (*n* = 21). The final study population included 6898 participants (2237 cases and 4661 controls).

Participants provided informed consent, and ethical approval was obtained from the Regional Ethics Review Board at Karolinska Institutet, Stockholm, Sweden (DNR 2006/476-31/4).

### Exposure

Overall tea consumption was assessed in the FFQ by asking how many cups per day or week the participant usually consumed of tea and herbal tea (örtte in Swedish, Supplementary Figure S1). Missing data for the tea variables (44.8% for tea and 82.6% for herbal tea) were imputed based on the zero-consumption assumption [17].

Total tea consumption was obtained by combining tea and herbal tea consumption. The total tea variable was analyzed as continuous and as categorical, while in a separate analysis tea and herbal tea consumptions were analyzed only as categorical variables. Tea and total tea consumptions were categorized as no consumer (0 cups/day), irregular consumer (< 1 cup/day), regular consumer (1–< 2 cups/day), and high consumer (≥2 cups/day), while herbal tea consumption was categorized as no consumer (0 cups/day), irregular consumer (< 1 cup/day), and regular consumer (≥1 cup/day).

### Covariates

The EIRA questionnaire contains several questions regarding lifestyle factors, which allowed to adjust for multiple covariates in addition to the matching factors. Smoking was categorized as never, former, irregular smoker, current smokers with < 20 pack-years, and current smokers with ≥20 pack-years. Body mass index (BMI) was categorized as underweight (< 18.5 kg/m^2^), normal weight (18.5–24.9 kg/m^2^), overweight (25–29.9 kg/m^2^), or obese (≥ 30 kg/m^2^). Education was categorized as < 10 years, 10–12 years, and > 12 years of education. Alcohol intake was categorized as ≤13 g/week, 14–40 g/week, 41–88 g/week, or ≥ 89 g/week (where 15 g of alcohol is approximately 1 glass of wine). Anti-citrullinated protein antibody (ACPA) status was measured in sera collected at RA diagnosis and defined as testing positive for anti-CCP2 antibodies (> 25 arbitrary units/mL), using the commercial anti-CCP2 assay from Eurodiagnostica.

### Statistical analyses

Descriptive baseline characteristics were summarized using percentages, means, and standard deviations by categories of total tea consumption, separately for cases and controls.

The dose-response trend of the association between tea consumption and the risk of RA was estimated using a restricted cubic spline with knots at 0, 0.29, and 2.29 cups per day.

Crude odds ratios (ORs) and their 95% confidence intervals (CIs) were estimated using conditional logistic regression that accounts for the matching factors age, sex, and residential area. We also performed multivariable analyses adjusted for smoking status, coffee, alcohol consumption, educational level, and BMI to estimate adjusted ORs (adjORs). Additional analyses for overall tea consumption were conducted stratified by sex, smoking status, and coffee consumption, and the category < 1 cup/day was used as a reference. The analyses stratified by smoking status and coffee consumption were based on an unconditional logistic model, additionally adjusted for the matching factors.

To evaluate the influence of the zero-consumption assumption on the main results, we performed two sensitivity analyses. First, we performed a complete case scenario analysis, in which all participants with missing values were excluded from the analyses. We then also performed a second sensitivity analysis in which 70% of the missing randomly were imputed as zero, while the remaining 30% were randomly allocated to the other three categories of total tea consumption.

All analyses were implemented in SAS (version 9.4), except the restricted cubic spline analyses, for which Stata (version 16.1) was used.

## Results

A total of 2237 cases, diagnosed during 2005–2018, and 4661 controls have been included in this study (Table 1). Controls were more likely to be high consumers (≥2 cups/day) of tea compared to RA cases (22.1% in controls vs. 19.7% in cases). Tea drinkers were less likely to smoke at diagnosis compared to non-tea drinkers, and as expected, there were more current smokers among the cases than among the controls (22.5% in cases vs. 13.8% in controls). Tea drinkers also had a lower intake of alcohol compared to non-tea drinkers and were more likely to not drink coffee.

**Table 1:** Baseline characteristics of patients with RA (cases, *n* = 2237) and controls (*n* = 4661) included in the EIRA study between 2005 and 2018, by categories of tea consumption

	RA cases				Controls			
Tea, cups/day (median)	Non-consumers (0 cups/day)	Irregular consumers (< 1 cup/day)	Regular consumers (1–2 cups/day)	High consumers (≥2 cups/day)	Non-consumers (0 cups/day)	Irregular consumers (< 1 cup/day)	Regular consumers (1–2 cups/day)	High consumers (≥2 cups/day)
**Tea, median cups/day**	**0**	**0.29**	**1**	**2**	**0**	**0.29**	**1**	**2.29**
***N***	955	453	388	441	1941	847	845	1028
**Female,** ***n*** **(%)**	582 (60.94%)	358 (79.03%)	288 (74.23%)	359 (81.41%)	1255 (64.66%)	643 (75.91%)	622 (73.61%)	817 (79.47%)
**Age, mean (std)**	56 (13)	51 (15)	56 (15)	55 (14)	55 (14)	51 (14)	56 (13)	54 (14)
**Coffee,** ***n*** **(%)**								
**0 cups/day**	70 (7.33)	44 (9.71)	43 (11.08)	100 (22.68)	172 (8.86)	79 (9.33)	83 (9.82)	269 (26.17)
**≤2 cups/day**	243 (25.45)	181 (39.96)	163 (42.01)	215 (48.75)	529 (27.25)	333 (39.32)	373 (44.14)	459 (44.65)
**2–3 cups/day**	222 (23.25)	101 (22.30)	85 (21.91)	74 (16.78)	479 (24.68)	211 (24.91)	204 (24.14)	148 (14.40)
**> 3 cups/day**	420 (43.98)	127 (28.04)	97 (25.00)	52 (11.79)	761 (39.21)	224 (26.45)	185 (21.89)	152 (14.79)
**Smoking,** ***n*** **(%)**								
**Never**	290 (30.37)	164 (36.20)	142 (36.60)	213 (48.30)	859 (44.26)	436 (51.48)	433 (51.24)	563 (54.77)
**Former**	310 (32.46)	160 (35.32)	145 (37.37)	138 (31.29)	564 (29.06)	245 (28.93)	257 (30.41)	262 (25.49)
**Current < 20 pack-years**	130 (13.61)	48 (10.60)	29 (7.47)	37 (8.39)	173 (8.91)	56 (6.61)	41 (4.85)	55 (5.35)
**Current ≥ 20 pack-years**	166 (17.38)	41 (9.05)	30 (7.73)	22 (4.99)	198 (10.20)	40 (4.72)	37 (4.38)	44 (4.28)
**Education,** ***n*** **(%)**								
**< 10 years**	253 (26.49%)	83 (18.32%)	66 (17.01%)	61 (13.83%)	401 (20.66%)	87 (10.27%)	123 (14.56%)	93 (9.05%)
**10–12 years**	263 (27.54%)	105 (23.18%)	95 (24,48%)	82 (18.59%)	552 (28.44%)	226 (26.68%)	205 (24.26%)	188 (18.29%)
**> 12 years**	439 (45.97%)	265 (58.59%)	227 (58.51%)	298 (67.57%)	988 (50.90%)	534 (63.05%)	517 (61.18%)	747(72.67%)
**BMI, mean (std)**	26.19 (4.55)	25.56 (4.91)	25.46 (4.63)	25.25 (4.39)	25.83 (4.35)	25.18 (4.42)	25.29 (6.76)	25.02 (4.26)
**Alcohol intake, mean grams/week (std)**	71.62 (101.94)	57.70 (68.60)	57.43 (70.53)	55.68 (92.35)	73.64 (95.06)	75.13 (120.05)	67.20 (75.58)	59.08 (74.42)

**Fig. 1:**
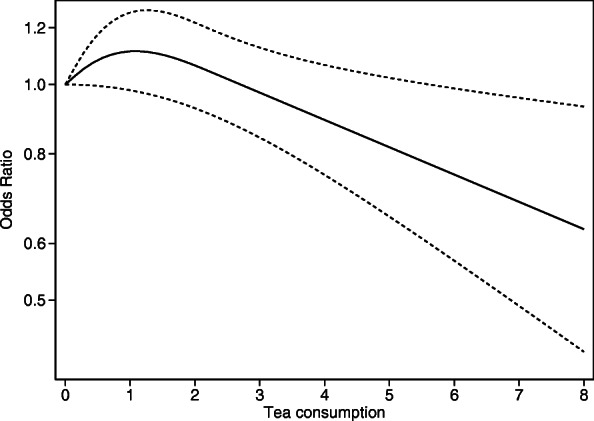
Dose-response odds ratio for the risk of rheumatoid arthritis (RA) by tea consumption. Adjusted for smoking, alcohol intake, coffee intake, educational level, and body mass index, in addition to the matching factors (age, sex, and residential area). Dotted lines correspond to 95% confidence intervals

The association between tea consumption and the risk of developing RA was not linear (coefficient of the second spline < 0.05), although a decrease in odds was evident for consumptions of 1 or more cups of tea per day (Fig. 1).

The crude odds of developing RA was 22% lower (OR = 0.78, 95% CI 0.66–0.92) among high consumers compared to irregular consumers (< 1 cup/day). After adjustment for education, BMI, smoking, alcohol intake, and coffee consumption, this inverse association was no longer statistically significant (adjOR = 0.85, 95% CI 0.71–1.01). There were also statistically significant lower odds for no tea consumption (adjOR = 0.82, 95% CI 0.70–0.95), although there was no difference between no consumption and high consumption of tea (adjOR = 0.97, 95% CI 0.83–1.13). A similar association was observed among women, but not among men. The full results from the association analysis are presented in Table 2.

**Table 2:** Odds ratios for the risk of rheumatoid arthritis by categories of overall tea consumption, overall, stratified by sex, ACPA, and smoking status, and among coffee drinkers among 2237 cases and 4661 controls diagnosed with RA during 2005–2018 in Sweden

	Overall tea consumption			
	Non-consumers (0 cups/day)	Irregular consumers (< 1 cup/day)	Regular consumers (1–2 cups/day)	High consumers (≥2 cups/day)
**Overall**				
Number	955/1941	453/847	388/845	441/1028
OR crude*	0.89 (0.77–1.03)	Ref	0.84 (0.71–1.00)	0.78 (0.66–0.92)
OR adjusted^±^	0.82 (0.70–0.95)	Ref	0.87 (0.73–1.04)	0.85 (0.71–1.01)
**Female**				
Number	582/1255	358/643	288/622	359/817
OR crude	0.82 (0.70–0.97)	Ref	0.82 (0.68–1.00)	0.79 (0.65–0.95)
OR adjusted	0.74 (0.62–0.88)	Ref	0.86 (0.70–1.05)	0.86 (0.70–1.04)
**Male**				
Number	373/686	95/204	100/223	82/211
OR crude	1.09 (0.82–1.46)	Ref	0.92 (0.65–1.31)	0.76 (0.52–1.10)
OR adjusted	1.04 (0.77–1.41)	Ref	0.95 (0.66–1.37)	0.79 (0.54–1.18)
**ACPA positive**				
Number	637/1156	321/527	268/501	277/630
OR crude	0.90 (0.76–1.07)	Ref	0.87 (0.71–1.07)	0.71 (0.58–0.87)
OR adjusted	0.81 (0.67–0.97)	Ref	0.88 (0.71–1.09)	0.76 (0.62–0.94)
**ACPA negative**				
Number	312/571	129/221	119/251	162/288
OR crude	0.91 (0.70–1.19)	Ref	0.80 (0.58–1.09)	0.97 (0.72–1.31)
OR adjusted	0.87 (0.66–1.14)	Ref	0.87 (0.63–1.19)	1.07 (0.78–1.46)
**Never smokers**				
Number	290/859	164/436	142/433	213/563
OR crude	0.91 (0.73–1.15)	Ref	0.90 (0.69–1.17)	1.03 (0.81–1.32)
OR adjusted	0.90 (0.71–1.13)	Ref	0.89 (0.68–1.16)	1.05 (0.82–1.35)
**Current smokers**				
Number	296/371	89/96	59/78	59/99
OR crude	0.84 (0.60–1.18)	Ref	0.84 (0.54–1.33)	0.63 (0.40–0.98)
OR adjusted	0.82 (0.58–1.16)	Ref	0.82 (0.51–1.30)	0.60 (0.38–0.95)
**Coffee drinkers**				
Number	885/1769	409/768	345/762	341/759
OR crude	0.93 (0.80–1.08)	Ref	0.84 (0.70–1.00)	0.83 (0.70–0.99)
OR adjusted	0.86 (0.74–1.00)	Ref	0.86 (0.72–1.03)	0.88 (0.74–1.06)

*The crude OR was based on a conditional logistic model where cases and controls were matched by age, sex, and residential area. The analyses for ACPA status were based on an unconditional logistic regression model, and the crude model was adjusted for the matching factors

^±^The model was additionally adjusted for coffee consumption, smoking status, alcohol, body mass index, and educational level

There was a statistically significant lower odds to develop ACPA-positive RA among the high tea consumers compared to irregular tea drinkers (adjOR = 0.76, 95% CI 0.62–0.94), but not to develop ACPA-negative RA.

The lowest odds ratio was observed among current smokers (adjOR = 0.60, 95% CI 0.38–0.95), while there was no association among never smokers. There was an inverse association between tea consumption and the risk of RA among smokers only for ACPA-positive RA (adjOR = 0.47, 95% CI 0.27–0.83), but not for ACPA-negative RA (adjOR = 1.39, 95% CI 0.53–3.66). Results restricted to coffee drinkers were similar to the main results (adjOR of high vs. irregular consumption = 0.88, 95% CI 0.74–1.06).

Regular consumers of herbal tea (≥1 cup/day) had a borderline statistically significant decreased odds to develop RA compared to irregular consumers of herbal tea (< 1 cup/day) (adjOR = 0.77, 95% CI 0.58–1.02) (Table 3).

**Table 3:** Odds ratios for the risk of developing rheumatoid arthritis by categories of tea consumption, stratified by type of tea among 2237 cases and 4661 controls, included in the EIRA study 2005–2018

	Numbers	Crude OR*	Adjusted OR^±^
**Herbal tea (cups/day)**			
No consumers (0)	1923/3959	0.94 (0.77–1.15)	0.86 (0.70–1.05)
Irregular consumers (< 1)	167/332	Ref	Ref
Regular consumers (≥1)	147/370	0.78 (0.59–1.03)	0.77 (0.58–1.02)
**Tea (cups/day)**			
No consumers (0)	1048/2150	0.89 (0.78–1.03)	0.83 (0.71–0.96)
Irregular consumers (< 1)	432/815	Ref	Ref
Regular consumers (1–< 2)	397/848	0.86 (0.72–1.02)	0.90 (0.76–1.08)
High consumers (≥2)	360/848	0.78 (0.66–0.94)	0.85 (0.71–1.03)

*The crude OR was based on a conditional logistic model where cases and controls were matched by age, sex, and residential area

^±^The model was additionally adjusted for coffee consumption, smoking status, alcohol, body mass index, and educational level

Sensitivity analyses were performed to assess the influence of the zero-assumption imputation of the missing values of the tea variables on the main result. In our first sensitivity analysis, a complete case scenario analysis was conducted, in which all participants with missing tea consumption were excluded (40.33% among controls, 40.86% among cases; Supplementary table S1). The adjusted odds ratio for high consumption compared to irregular consumption was 0.83 (95% CI 0.70–0.98) and statistically significant (Table 4). The odds ratio for non-tea consumers was increased, but not statistically significant (adjOR 1.26; 95% CI 0.83–1.93). In our second sensitivity analysis, 70% of the missing were randomly imputed as no consumption, while the remaining 30% were randomly assigned to the other categories (10% per category). The results from this second analysis were similar to those from the main analysis, although the OR in the no consumption category was not significant (adjOR = 0.88, 95% CI 0.76–1.02) while the OR in the high consumption category was statistically significant (adjOR = 0.85, 95% CI 0.73–0.99).

**Table 4:** Odds ratios for the risk of developing rheumatoid arthritis according to two sensitivity analyses

	No consumption (0 cups/day)	Irregular consumption (< 1 cup/day)	Regular consumption (1–2 cups/day)	High consumption (≥2 cups/day)
	Complete case analysis			
Number cases/controls	41/61	453/847	388/845	441/1028
OR adjusted^±^	1.26 (0.82–1.92)	Ref	0.87 (0.73–1.03)	0.83 (0.70–0.98)
	Random allocation of missing (70% null category, 10% in each of the others)			
Number cases/controls	687/1369	551/1046	473/1022	526/1224
OR adjusted^±^	0.88 (0.76–1.02)	Ref	0.89 (0.76–1.04)	0.85 (0.73–0.99)

^±^The model was adjusted for coffee consumption, smoking status, alcohol, body mass index, and educational level, in addition to the matching factors (age, sex, and residential area)

## Discussion

In this large population-based case-control study, a lower odds of developing ACPA-positive RA was observed among high tea consumers (≥2 cups per day) as compared to irregular tea drinkers (< 1 cup per day). This association was strongest among current smokers, for whom high tea consumption reduced the odds of developing RA by almost half compared to irregular tea drinkers.

Analyses from the sensitivity analyses confirmed the robustness of the lower odds of RA observed among those who drank ≥ 2 tea cups/day. However, the observed inverse association among the no tea consumers compared to irregular tea consumers was not confirmed in the sensitivity analyses, thus suggesting that this result might have been affected by bias due to misclassification in our main analysis.

Previous studies on tea consumption and the risk of developing RA have found conflicting results. A cohort study found no association between consumption of > 3 cups/day compared to no tea consumption using data from the Nurse’s Health Study, with 480 confirmed cases of RA out of 83,124 women [10]. An analysis of 185 confirmed cases of RA out of 76,853 women from the Women’s Health Initiative Observational Study showed an increased risk of incident RA among ever tea consumers compared to no tea consumers [12]. Furthermore, tea consumption was inversely associated with RA development in the Iowa Women’s Health Study [11]. However, the reduced risk observed among women consuming > 3 cups/day compared to non-consumers in the Iowa Women’s Health Study was based on a modest sample size of 5 observed RA cases. A case-control study, also with a limited number of cases and controls, showed no association between tea and inflammatory polyarthritis [13], while another case-control study showed an inverse association between green tea consumption and RA [14]. The three cohort studies with conflicting results have all been conducted in the USA using a similar design. However, tea consumption varies not only between, but also within countries and across cultures, which highlights the difficulty of studying these modifiable dietary factors. The heterogeneity of the studies, and the small sample size, was further discussed by Lee et al. in a meta-analysis, with the conclusion that further studies were needed [15].

Our present study, the so-far largest case-control study on tea consumption in RA, contributes to this literature with data on tea consumption for over 2200 RA cases. According to sales records in Sweden, 62% of tea buyers consume black tea, 16% green tea, 5% red tea, and 17% herbal tea. All kinds of tea are rich in polyphenols, but depending on the degree of fermentation, the type of polyphenol varies. Green tea is rich in the polyphenol group called catechins, such as epigallocatechin-3-gallate (EGCG), and several studies on EGCG have shown an inverse association between tea consumption and the risk and progression of RA [18]. Black tea, however, only contains 30% of the catechin amount found in green tea, but is rich in the polyphenols thearubigins and theaflavin s[19], which can reduce inflammation and cartilage erosion [20]. Moreover, the antioxidant properties of the polyphenols have been shown to be associated with a decreased risk of RA [21, 22]. In our present study, tea consumption was particularly associated with a decreased risk of RA among smokers and in the ACPA-positive subset of RA. Interestingly, previous studies have showed that flavonoids can decrease the risk of overall mortality and cancer, particularly among smokers. Although the biological mechanism behind this association is yet unclear, it is of interest to note that this observation is in line with the findings in our present study [23, 24]. The restriction of the potential protective effects to the ACPA-positive subset of RA might indicate that high tea consumption had a preferential effect on the adaptive immune response [25].

The main strength of this study is its high participation rate (90% of cases and 69% of controls) and design: the EIRA study is one of the world’s largest population-based case-control studies including incident cases of RA. Its baseline questionnaire contains extensive questions about several environmental factors, making it possible to account for many potential confounders. However, our study has also some limitations. There was a large percentage of missing for tea consumption. To account for this, we assumed that most, if not all, of them corresponded to a no tea consumption, based on several factors. First of all, the overall questionnaire in general and the FFQ in particular was extensive to fill in, and, in regard to tea consumption, the participant had to write the numbers of cups/glasses of more than 30 different items (see Supplementary figure S1). It is reasonable to assume that some participants might have filled in the FFQ for only the items that were consumed, leaving the others blank. This hypothesis was also supported by the very limited proportion of patients that did fill in all the variables (2.6%). Moreover, based on statistics for Swedish consumption of tea, we expected around 50% of non-tea consumers in our population [26], a percentage even higher than what we obtained after imputing all non-entries as zero (42%). To overcome this ascertainment limitation, we instead used irregular tea consumption as a reference. Our sensitivity analyses showed the results for regular vs. irregular drinkers to be robust. However, it also suggested that the no consumption category might have been affected by some bias, thus refraining us from drawing a conclusion about those participants. Another limitation of this study is the possibility of recall bias, due to the case-control design based on self-report. However, we do not believe that recall bias has influenced the amount of missing, since the percentage was similar among cases (40.86%) and controls (40.33%). As recommendations on tea consumption are not available in RA and tea consumption after RA diagnosis is unlikely to change [27] and the EIRA questionnaire also is filled in during the immediate weeks after diagnosis, we do not believe reverse causation affects our results. This assumption is strengthened by the fact that the inverse association between high tea consumption and risk for RA was seen only in the ACPA-positive subset of RA and the fact that included individuals did not know their ACPA status when answering the questionnaires. Furthermore, the proportion of people reporting consumption of herbal tea was low, and this estimate might thus be unreliable. However, it should be noted that the proportion of participants that reported tea consumption was indeed in line with sales statistics for tea in Sweden.

## Conclusion

This study showed an inverse association with the risk of RA among high tea consumers compared to irregular consumers among smokers and in the ACPA-positive subset of RA. We could not draw any conclusion about no tea consumption and the risk of RA, due to the possibility of bias.

## Supplementary Information

**Additional file 1: Supplementary Figure S1**. section of the EIRA questionnaire containing the tea questions (in Swedish). **Supplementary table S1**. Baseline characteristics of patients with RA (cases, n = 1323) and controls (n = 2781) included in the EIRA study between 2005-2018 after excluding individuals with missing tea consumption, by categories of tea consumption

## Data Availability

Due to the content of the ethical approval and consents, data from EIRA cannot be publicly shared. Please contact the principal investigators for data requests for applicable studies. For further information, go to http://www.eirasweden.se/Kontakt_EIRA.htm.
