# Supplementary material for: Is tea consumption associated with reduction of risk of rheumatoid arthritis? A Swedish case-control study
Source: Arthritis Res Ther. 2021 Aug 7;23:209. doi: 10.1186/s13075-021-02583-y (PMC8349003; doi:10.1186/s13075-021-02583-y)
Supplement: Supplementary file 1 — Additional file 1: Supplementary Figure S1. section of the EIRA questionnaire containing the tea questions (in Swedish). Supplementary table S1. Baseline characteristics of patients with RA (cases, n = 1323) and controls (n = 2781) included in the EIRA study between 2005-2018 after excluding individuals with missing tea consumption, by categories of tea consumption [file 13075_2021_2583_MOESM1_ESM.docx]

**Online Supplementary material**

This file contains online support data for Westerlind et al ”Is tea consumption associated with reduction of risk in rheumatoid arthritis? A Swedish case control study”

Items included:

* Supplementary Figure S1

* Supplementary Table S1

**Supplementary Figure S1**: section of the EIRA questionnaire containing the tea questions (in Swedish)


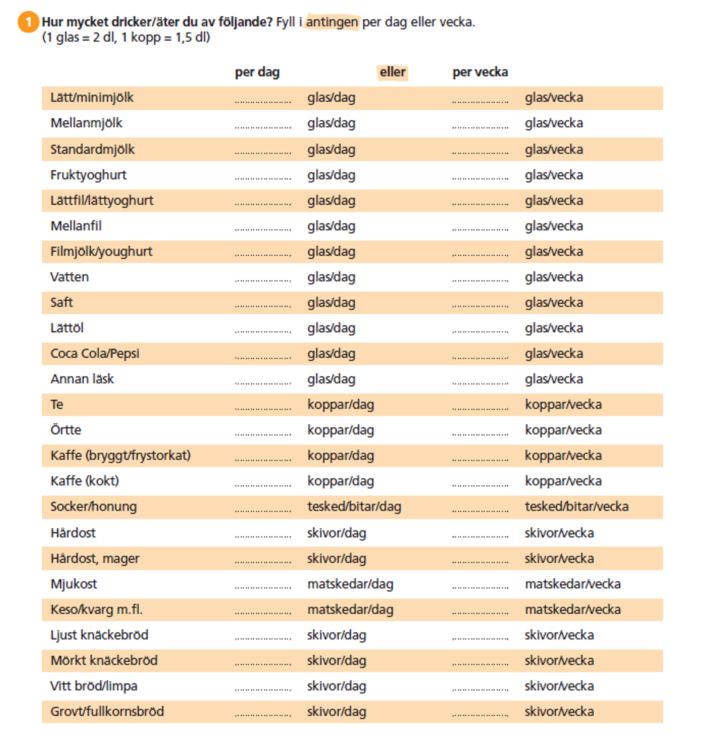


**Supplementary table S1**. Baseline characteristics of patients with RA (cases, n = 1323) and controls (n = 2781) included in the EIRA study between 2005-2018 after excluding individuals with missing tea consumption, by categories of tea consumption

|  | **RA cases** | | | | **Controls** | | | |
| --- | --- | --- | --- | --- | --- | --- | --- | --- |
| **Tea, cups/day (median)** | **Non consumers  (0 cups/day)** | **Irregular consumers (<1 cup/day)** | **Regular consumers (1-2 cups/day)** | **High consumers (≥2 cups/day)** | **Non consumers  (0 cups/day)** | **Irregular consumers (<1 cup/day)** | **Regular consumers (1-2 cups/day)** | **High consumers (≥2 cups/day)** |
| **Tea, median cups/day** | **0** | **0.29** | **1** | **2** | **0** | **0.29** | **1** | **2.29** |
| **N** | 41 | 453 | 388 | 441 | 61 | 847 | 845 | 1028 |
| **Female, n (%)** | 25 (60.98%) | 358 (79.03%) | 288 (74.23%) | 359 (81.41%) | 44 (72.13%) | 643 (75.91%) | 622 (73.61%) | 817 (79.47%) |
| **Age, mean (std)** | 51 (15) | 51 (15) | 56 (15) | 55 (14) | 55 (13) | 51 (14) | 56 (13) | 54 (14) |
| **Coffee, n (%)** |  |  |  |  |  |  |  |  |
| **0 cups/day** | 9 (21.95) | 44 (9.71) | 43 (11.08) | 100 (22.68) | 2 (3.28) | 79 (9.33) | 83 (9.82) | 269 (26.17) |
| **≤2 cups/day** | 10 (24.39) | 181 (39.96) | 163 (42.01) | 215 (48.75) | 17 (27.87) | 333 (39.32) | 373 (44.14) | 459 (44.65) |
| **2-3 cups/day** | 6 (14.63) | 101 (22.30) | 85 (21.91) | 74 (16.78) | 18 (29.51) | 211 (24.91) | 204 (24.14) | 148 (14.40) |
| **>3 cups/day** | 16 (39.02) | 127 (28.04) | 97 (25.00) | 52 (11.79) | 24 (39.34) | 224 (26.45) | 185 (21.89) | 152 (14.79) |
| **Smoking, n (%)** |  |  |  |  |  |  |  |  |
| **Never** | 19 (46.34) | 164 (36.20) | 142 (36.60) | 213 (48.30) | 26 (42.62) | 436 (51.48) | 433 (51.24) | 563 (54.77) |
| **Former** | 11 (26.83) | 160 (35.32) | 145 (37.37) | 138 (31.29) | 18 (29.51) | 245 (28.93) | 257 (30.41) | 262 (25.49) |
| **Current <20 pack-years** | 3 (7.32) | 48 (10.60) | 29 (7.47) | 37 (8.39) | 5 (8.20) | 56 (6.61) | 41 (4.85) | 55 (5.35) |
| **Current ≥20 pack-years** | 6 (14.63) | 41 (9.05) | 30 (7.73) | 22 (4.99) | 5 (8.20) | 40 (4.72) | 37 (4.38) | 44 (4.28) |
| **Education, n (%)** |  |  |  |  |  |  |  |  |
| **<10 years** | 9 (21.95) | 83 (18.32%) | 66 (17.01%) | 61 (13.83%) | 8 (13.11) | 87 (10.27%) | 123 (14.56%) | 93 (9.05%) |
| **10–12 years** | 14 (34.15) | 105 (23.18%) | 95 (24,48%) | 82 (18.59%) | 20 (32.79) | 226 (26.68%) | 205 (24.26%) | 188 (18.29%) |
| **>12 years** | 18 (43.90) | 265 (58.59%) | 227 (58.51%) | 298 (67.57%) | 33 (54.10) | 534 (63.05%) | 517 (61.18%) | 747(72.67%) |
| **BMI, mean (std)** | 26.98 (5.97) | 25.56 (4.91) | 25.46 (4.63) | 25.25 (4.39) | 24.74 (3.95) | 25.18 (4.42) | 25.29 (6.76) | 25.02 (4.26) |
| **Alcohol intake, mean grams/week (std)** | 60.93 (66.01) | 57.70 (68.60) | 57.43 (70.53) | 55.68 (92.35) | 87.30 (102.19) | 75.13 (120.05) | 67.20 (75.58) | 59.08 (74.42) |
